# A *Moringa oleifera*-based formulation for growth and mitigation of drought stress in tomato plants

**DOI:** 10.3389/fpls.2026.1771042

**Published:** 2026-03-12

**Authors:** Abderezak Berouaken, Cosima Damiana Calvano, Angelica Schena, Simone Pascuzzi, Francesco Paciolla, Claudio Cocozza, Pasquale Losciale, Vito Ugenti, Giuseppe di Leo, Antonella Simini, Ornella Incerti, Michela Cecchin, Alice Baroni, Antonio Ippolito, Chiara Pituello, Simona Marianna Sanzani

**Affiliations:** 1 Dipartimento di Scienze del Suolo, della Pianta e degli Alimenti, Università degli Studi di Bari Aldo Moro, Bari, Italy; 2 Dipartimento di Chimica, Università degli Studi di Bari Aldo Moro, Bari, Italy; 3 SICIT Group S.p.A., Chiampo, VI, Italy

**Keywords:** bioactive compounds, horticultural crops, plant extract, sustainability, water deficiency

## Abstract

**Introduction:**

Contemporary agriculture is undergoing a profound transformation aiming at reducing environmental pollution, improving food security, and tackling the climate crisis. To respond to these challenges, the European Commission launched in 2020 the Green Deal initiative, which has among its main goals for 2030 a reduction in the use of synthetic fertilizers and plant protection products, as well as an increase in organic cultivation. In the last three decades biostimulants of natural origin have contributed to reduce agriculture footprint.

**Methods:**

The objective of the present research was to test the efficacy of a new formulation based on a hydrolysed *Moringa oleifera* aqueous extract in presence and absence of a stress (drought). The assays were carried out on tomato plants grown in a greenhouse. The tested formulation was applied either by foliar spray or soil drenching. The abiotic stress was induced by suspending irrigation until wilting point. Efficacy was assessed by measuring plant vegetative parameters as height, Normalised Difference Vegetation Index (NDVI), photosynthetic pigment content, and Relative Water Content (RWC), as well as production parameters as fruit set and cluster weight.

**Results and discussion:**

The results highlighted an overall positive effect of the tested formulation on vegetative parameters when applied by spraying in absence of stress; however, a different behaviour was observed as far as the resistance against drought concerns, being the *Moringa* formulate more effective as soil drenching. The positive effect could be ascribed to the presence in the formulate of numerous bioactive molecules, including polyphenols, flavonoids, organic acids, amino-acid derivatives, nucleosides, lipids, and bioactive peptides. Although further in the field large-scale trials are needed, these preliminary data support the use of the formulation in sustainable agriculture.

## 1 Introduction

The growing demand by consumers for agricultural products derived from sustainable cultivation systems has recently led to considerable interest in biostimulants, which are organic materials, other than fertilisers, that enhance plant growth and development when applied in minute quantities (Kauffman et al., 2007). Their components include microorganisms, plant and algae extracts, amino acids, humic substances, mineral salts, and some chemicals with biostimulant properties (EBIC, 2025). These substances can be applied to the plant and/or to the rhizosphere throughout the crop cycle; they stimulate plant nutritional processes independently of the nutrient content, with the aim of improving one or more of the following characteristics of the plant or its rhizosphere: nutrient use efficiency, tolerance to abiotic stress, qualitative characteristics, nutrient availability in the soil or rhizosphere (European Commission, 2019; Calvo et al., 2014). However, it has been shown that treatment with biostimulants increases the expression of stress-tolerant genes, improving the general plant’s response to stresses including even biotic stresses (Varun & Lakshmi, 2025).

*Moringa oleifera* L. (synonym: *Moringa pterygosperma* Gaertner) is a tree belonging to the *Moringaceae* family that grows from Northwest Pakistan to North India. The tree is deciduous, well adapted to poor soils and drought. It has been introduced in several geographical areas like Afghanistan, Bangladesh, Sri Lanka, Africa, West Asia and the Americas, from Mexico to Peru, Caribbean Islands, Paraguay, and Brazil (Ferreira et al., 2008). Extracts from all parts of the plant have several beneficial properties, recognized by common use and documented by the literature. For example, the leaves have a rich composition of essential inorganic nutrients, proteins, vitamins, sugars, fibres, phenolics, and free proline, as well as important amounts of phytohormones, including auxins, cytokinins, and gibberellins (Mashamaite et al., 2022). Recently, potential inducers of plant resistance to stresses were identified in aqueous extracts of *M. oleifera* leaves by Admane et al. (2023). However, the extraction process and the solvents used to separate the various components, as well as the geographical area and the plant cultivation technique, might influence the final composition of the extract (Admane et al., 2023; Pusta and Macusi, 2024). *M. oleifera* extract might undergo different processing steps to increase the range of bioactive available compounds, including hydrolysis. The latter could be carried out on the whole extract or its fraction, for example the protein one. Hydrolysis can be obtained chemically or microbially, and hydrolysates are particularly rich in low-molecular-weight components, including peptides, free amino acids, vitamins, and trace elements, whereas partial lignin breakdown increases the content in phenolic compounds (Mitchell et al., 2014). Plant hydrolysates have been identified to improve the performance of several horticultural crops, including increased shoot and root biomass, and their productivity (Colla and Rouphael, 2015).

Tomato (*Solanum lycopersicum* L.) is a species belonging to the botanical family of *Solanaceae*, that was imported from the Andes region into Europe in the 16^th^ century (Bergougnoux, 2014). It is one of the most cultivated and consumed vegetables. World tomato production in 2023 was 192 million tonnes, covering an area of 5.4 million ha, with China as the world’s leading producer, followed by India, Turkey, USA, and Egypt; Italy ranked sixth in the world, and first in Europe, with a production of 6 million t (FAOSTAT, 2023). Tomato is consumed fresh but also processed into a variety of products as sauces, juices, purees. As such, its cultivation and postharvest handling is of paramount importance, and the search for sustainable strategies to increase its productivity reducing losses and wastes is continuous. These latter are primarily caused by other living organisms, including insects, bacteria, fungi, and weeds, that negatively affect plant growth and productivity. Secondly, abiotic stresses, particularly drought and nutrient deficiencies, represent major challenges, particularly in developing countries (Bulgari et al., 2019); as such, the application of biostimulants in tomato cultivation represents an effective agronomic strategy to enhance tolerance to abiotic stresses. These stress factors negatively affect key physiological and biochemical processes, including photosynthetic activity, nutrient uptake, cellular redox homeostasis, and water relations, ultimately leading to reductions in plant growth and yield (Kadoglidou et al., 2025). In tomato, biostimulant treatments have been shown to enhance antioxidant enzyme activity, improve osmotic adjustment through the accumulation of compatible solutes, regulate ion homeostasis under saline conditions, and increase water use efficiency during drought stress (Bulgari et al., 2019). Moreover, they can influence gene expression related to stress signaling and hormone‐like activity, thereby reinforcing plant adaptive responses to unfavourable environments (Rouphael and Colla, 2020). Collectively, these mechanisms provide a strong scientific rationale for the use of biostimulants as sustainable tools to mitigate abiotic stress impacts in tomato cultivation, particularly under the increasing pressure of climate change. Existing evidence supports the potential of plant hydrolysates to improve tomato performance under abiotic stress (Francesca et al., 2021; Ceccarelli et al., 2021), but significant gaps remain in mechanistic understanding, formulation specificity, target-stress efficacy, and agronomic standardization. Addressing these gaps through targeted multi-disciplinary studies will be necessary to substantiate the potential use of novel protein-based extracts in tomato cultivation. The aim of the present investigation was to demonstrate the efficacy of a new formulation industrially produced based on a hydrolysed *M. oleifera* extract in improving tomato plant growth and response to drought stress with the aid of a multi-targeted approach from seed priming to the analysis of morphometric, physiological, and production parameters of the plants.

## 2 Materials and methods

### 2.1 Moringa extract

The applied treatment was a formulation based on an aqueous hydrolysed extract (MOF) of whole aerial part of *Moringa oleifera* (MOF) plants (SICIT Group S.p.A., Campo, VI, Italy). The extract was produced by a patented technology, specifically developed by SICIT (Patent Nr: WO2023187060A1). The *Moringa* plants used for the formulate come from a specifically developed industrial cultivation. Moreover, the product batch used for the experiments was analysed for qualitative composition as reported below.

### 2.2 Formulate composition analysis

#### 2.2.1 Chemicals

Water (H_2_O), acetonitrile (ACN), methanol (MeOH), sodium chloride (NaCl) used in the extraction process, and formic acid (LC-MS grade), were purchased from Merck (Milan, Italy). Standard solutions for negative and positive calibrations were purchased from Thermo Fisher Scientific (Waltham, MA, USA).

#### 2.2.2 Sample purification

The salting-out liquid-liquid extraction method (SALLE) described by da Silva Monteiro et al. (2025) was adapted. For the extraction, 1 g of the formulate lyophilized powder was added to 1 mL of ultrapure water and 2 mL of acetonitrile, and the mixture was vortexed for 90 s. Then, 0.25 g of NaCl was added to disperse the solvents. The mixture was vortexed for 3 min and centrifuged at 5000 *×g* for 10 min to separate the acetonitrile/water phases. The collected supernatant was subjected to solvent evaporation using a SpeedVac concentrator (Thermo Fisher Scientific). The extract was then resuspended in 0.25 mL of a 25% (v/v) methanol solution and filtered through a 0.45 μm membrane for injection.

#### 2.2.3 Instrumentation and operating conditions

Samples were analysed by an Ultimate 3000 UHPLC system (Thermo Fisher Scientific) coupled with a hybrid Q-Exactive mass spectrometer (Thermo Fisher Scientific), equipped with a heated electrospray ionization (HESI) source and a higher collisional energy dissociation (HCD) cell for tandem MS analyses. Total phenolics extracts were separated by reversed-phase liquid chromatography (RPLC) on a conventional ODS column packed with type B silica core–shell microparticles, Supelco Ascentis^®^ Express (150 × 2.1 mm ID, particle size 2.7 μm) equipped with a precolumn (50 × 2.1 mm ID) (Bellefonte, PA, USA). During RPLC analyses, the column temperature was maintained at 40 °C. Samples were injected into the chromatographic system using an autosampler with an injection volume of 5 μL. The mobile phases consisted of H_2_O (solvent A) and CH_3_CN (solvent B), both containing 0.1% formic acid (FA). The binary mobile phase gradient, operated at a constant flow rate of 200 μL/min, was as follows: 0–5 min isocratic at 5% solvent B; 5–8 min linear gradient from 5 to 15% v/v solvent B; 8–25 min linear gradient from 15 to 18% v/v solvent B; 25–28 min linear gradient from 18 to 40% v/v solvent B; 28–30 min linear gradient from 40 to 100% v/v solvent B; 30–40 min isocratic at 100% solvent B; 40–45 min gradient from 100 to 5% v/v solvent B; 45–50 min isocratic at 5% v/v solvent B. HESI source and optics parameters were kept constant during LC-MS analyses: sheath gas flow rate, 35 arbitrary units (au); auxiliary gas flow rate, 15 au; spray voltage, 3.5 kV (both in positive and negative ion mode); capillary temperature, 320 °C; S-Lens RF level, 100%. Scanning was performed over an *m/z* range from 80 to 1500 with a resolution of 70,000 in Full-MS and 17,500 in MS². An Ultimate 3000 Diode Array Detector (DAD) was used for the acquisition of UV–Vis spectra after RPLC separations. The following operating parameters were set: minimum acquisition wavelength: 190 nm; maximum acquisition wavelength: 800 nm; bunch width: 1 nm; data collection rate: 5 Hz; response time 2 s. LC-MS instrument control and data acquisition were carried out using the Xcalibur software (Thermo Fisher Scientific). MS/MS spectra were obtained in higher-energy collisional dissociation (HCD) mode under data-dependent acquisition conditions. The collision energy was set to 30 eV with an isolation window of 1 *m/z*. MS data were imported, processed, and finally converted into figures using SigmaPlot 14.0 software (Systat Software, London, UK), while ChemDraw Pro 8.0.3 (CambridgeSoft Corporation, Cambridge, MA, USA) was used to draw and interpret the chemical structures.

### 2.3 Tomato seeds priming assay

The germination rate of tomato seeds (*Solanum lycopersicum* cv. Thonyno, F1 hybrid) was evaluated in the presence of the MOF formulate at 3 mL/L. Seeds primed in water represented the control.

The seeds (45 seeds per treatment) were rinsed in sterile distilled water and left in contact with the solution (water or biostimulant formulation) for 24 h at 20 °C on a rotary shaker (110 rpm). After this period, the seeds were placed in sterile Petri dishes (3 replicates of 15 seeds per plate) containing 5 layers of sterile filter paper moistened with 15 mL of sterile distilled water. The plates were then incubated at 21 °C with 16/8 h of light/darkness. Germination process was monitored daily and expressed as percentage of germinated seeds over the total seeds. The length of the roots was also evaluated and expressed in cm.

### 2.4 In planta assays

Assay were performed on tomato plants (*S. lycopersicum* cv. DRW 7749, F1 hybrid with undetermined development), grown in 8 L plastic pots in a greenhouse located in Fasano (Brindisi province, Italy), in a randomised block design. The experiment was conducted applying the abiotic stress drought. Treatments were applied once at transplanting immersing roots for 3 min in a 7 mL/L MOF solution, and then periodically (every 10 days) as foliar spray or soil drenching. To perform foliar treatment, a 3 mL/L MOF solution was sprayed until dripping on the plants (approx. 10–30 mL according to plant size), wetting the leaves both at the abaxial and adaxial side. For fertigation, a standard amount of 200 mL of 3 mL/L MOF was administered to each plant. Control plants were sprayed/irrigated with the same amount of distilled water.

#### 2.4.1 Abiotic stress assay

Six groups were prepared, consisting of 15 plants each: T0) no water stress control (CTRL); T1) water stress control (STRESS); T2) foliar treatment with MOF (F-MOF); T3) foliar treatment with MOF combined with abiotic stress (F-MOF STRESS). T4) fertigation with MOF (S-MOF); T5) fertigation with MOF combined with abiotic stress (S-MOF STRESS).

The soil was analysed to determine its pH in water and in KCl, electrical conductivity, organic carbon content, and particle size distribution. The latter results were inserted into an online calculator (http://www.dynsystem.com/netstorm/soilwater.html) to detect the following hydrological parameters:

Wilting Point (WP), *i.e.* the water content at a matrix’s potential of -1,5 *MPa* (-15 bars), roughly corresponding to the lower limit of the available water (a_w_). It is expressed as water units/soil units.

Field Capacity (FC), *i.e.* is the water content at the upper limit of the a_w_. It roughly corresponds to a matrix’s potential of -0.03 *MPa* (-0.3 bars). It is expressed as water units/soil units.

Available Water (a_w_) = FC – WP. It is expressed as a percentage by volume (volume of water/volume of soil sample).

Bulk density (BD) = (1 - saturation) × 2.65, bulk density, or apparent molar property, represents the mass of soil per unit volume and is a function of texture and porosity.

Following indications from the literature (Nuruddin et al., 2003; Oancea et al., 2013), water stress was induced during the fruit set. This developmental phase is highly sensitive to water limitation and plays a key role in determining final fruit number and yield. Water stress was applied by not irrigating the plants until a soil moisture of 25% was reached, which corresponded to an approximately 80% depletion of the available soil water (Nuruddin et al., 2003). The soil moisture was measured using a Digital Soil Moisture Meter (YINMIK, Jinan City, China), which uses FDR technology to measure the difference in relative dielectric conductivity of soil (Qin et al., 2018). The measurements were validated using a TDR PICO64 instrument (IMKO, IMKO Micromodultechnik GmbH, Germany).

#### 2.4.2 Vegetative parameters

Before and after the application of the formulation, the height of each plant from the crown to the apical shoot was measured and expressed in cm.

The Normalized Difference Vegetation Index (NDVI), the most widely used vegetation indices to characterize vegetation cover and assess its level of vigour over time, was calculated as follows:


$$NDVI=\frac{\left(NIR-Red\right)}{\left(NIR+Red\right)}$$

where NIR represents the near-infrared reflectance and Red represents the red band reflectance. Healthy vegetation strongly reflects in the NIR region and absorbs in the red band due to photosynthesis activity (Sims and Gamon, 2003). The NDVI ranges between -1 and +1. Negative values correspond to non-vegetated or anthropic areas; values close to 0 denote almost absent or highly stressed plant cover; intermediate values (from 0.4 to 0.6) indicate moderate vegetation vigour and values close to 1 indicate very high vigorous vegetation. NDVI was obtained from multispectral images acquired using the MicaSense ALTUM-PT™ (AgEagle Aerial Systems Inc, Wichita, Kansas, USA) camera. It captured simultaneously six spectral bands centred at: Blue (475 ± 32 nm), Green (560 ± 27 nm), Red (668 ± 14 nm), Red Edge (717 ± 12 nm), and Near-IR (842 ± 57 nm). The camera was mounted on a tripod to ensure image stability. To override the influence of light exposure, at the beginning and at the end of each tomato row, the camera was radiometrically calibrated using a reflectance panel with a known reflectance value. Due to the proximity of the tomato plants from the camera (about 1.5 m), the multispectral bands acquired for each image were optically aligned to correctly reconstruct the binary mask of the plant and avoid inconsistencies in the calculation of the NDVI. This pipeline was performed in MATLAB2024a using a custom routine, and an NDVI map was generated for each plant. The measurements were carried out on days 0, 3, 7 and 11 from water stress application. For each treatment, NDVI values of three representative plants per treatment were calculated. The increase or decrease rate of NDVI (%) over time was computed using the following formula:


$$\text{NDVI}\;\text{rate}\;(\%)=\frac{\left(NDVI\text{t}-\text{NDVIi}\right)}{\text{NDVIi }\times 100}$$

where NDVI_t_ is the value recorded on the day t and NDVI_i_ is the initial value recorded at time zero.

#### 2.4.3 Determination of pigments’ content

The total chlorophyll and carotenoids contents were determined to test the plant’s response in terms of photosynthetic efficiency following treatment with the biostimulant. For each treatment, leaf samples were collected before and after the stress and stored at -80 °C until use. The pigments were initially extracted by acetone (Admane et al., 2023). Briefly, 3 leaves per plant were crushed with a mortar and a pestle, and 0.1 g were placed in 2 mL of 100% acetone. Three replicates were performed for each sample. The samples were incubated for 15 min on ice and then centrifuged at 14.000 *×g* for 5 min. Finally, the supernatant was filtered by a nylon syringe filter (0.45 µm, Merck). The absorbance reading was performed with Cary 60 UV-Vis Spectrophotometer (Agilent Technologies, Santa Clara, CA, USA). The samples were read at three different wavelengths: 662, 645, and 470 nm. The values obtained for each sample were entered into three different formulas (Admane et al., 2023) to obtain the pigment content:


$$\text{Chlorophyll a }({\text{Chl}}_{\text{a}})=(11.24-\text{A}662)\times (2.04-\text{A}645)$$

$$\text{Chlorophyll b }({\text{Chl}}_{\text{b}})=(20.13-\text{A}645)\times (4.19-\text{A}662)$$

$$\text{Carotenoids}=(1000-\text{A}470\times 1.9{\text{ Chl}}_{\text{a}}\times 63.14{\text{ Chl}}_{\text{b}})/214$$

#### 2.4.4 Leaf relative water content

The relative water content of the leaves was determined as measurement of its hydration status (Mullan and Pietragalla, 2012). Five young and well-developed leaves with 1 cm petiole were collected from three representative plants and weighed by an analytical balance (WLC 0.6/B1, Radwag, Radom Poland) to obtain the fresh weight (FW). The leaves were then placed in squared Petri plate (Aptaca, Canelli, Asti, Italy) containing 5 layers of sterile absorbent paper soaked by sterile distilled water and a net on which the leaves were laid. The net was used as a support to avoid direct contact between the leaf and the wet paper, leaving only the terminal part of the petiole into contact. The leaves were incubated at 4 °C for 30 h, until the cell turgor was reached. The leaves were re-weighed, and the turgid weight (TW) value was obtained. Finally, the leaves were placed in an incubator at 37 °C for 72 h until complete dehydration, for measuring the dry weight (DW). The three values obtained from each leaf have been included in the formula (Mullan and Pietragalla, 2012) of the relative water content:


$$\text{RWC}\;(\%)=\frac{\left(\text{FW }-\text{ DW}\right)}{\left(\text{TW}-\text{DW}\right)}\times \;100$$

The RWC measurements were carried out at the beginning and at the end of the stress.

#### 2.4.5 Production parameters

The yield was evaluated in terms of fruit set (number of developed fruits over the total number of flowers, %) and fruit cluster weight (g) measured by an analytical balance (Radwag).

### 2.5 Statistical analysis

The effect of treatments was expressed by a control index (CI) using the following formula: CI (%) = [(C − T)/C] × 100, where C and T represent the mean value of each parameter assessed in control and treated samples, respectively.

All data were subjected to a one-way analysis of variance (One-way ANOVA) using the statistical software package Statistics for Windows v7.0.61.0 (StatSoft, Tulsa, OK, USA). Means were separated utilising Duncan’s Multiple Range Test (DMRT) as a *post hoc* test, performed at a p < 0.05 significance level. Percentage data were subjected to arcsine square root transformation before ANOVA analysis.

## 3 Results and discussion

### 3.1 Formulation composition

**Figure 1:**
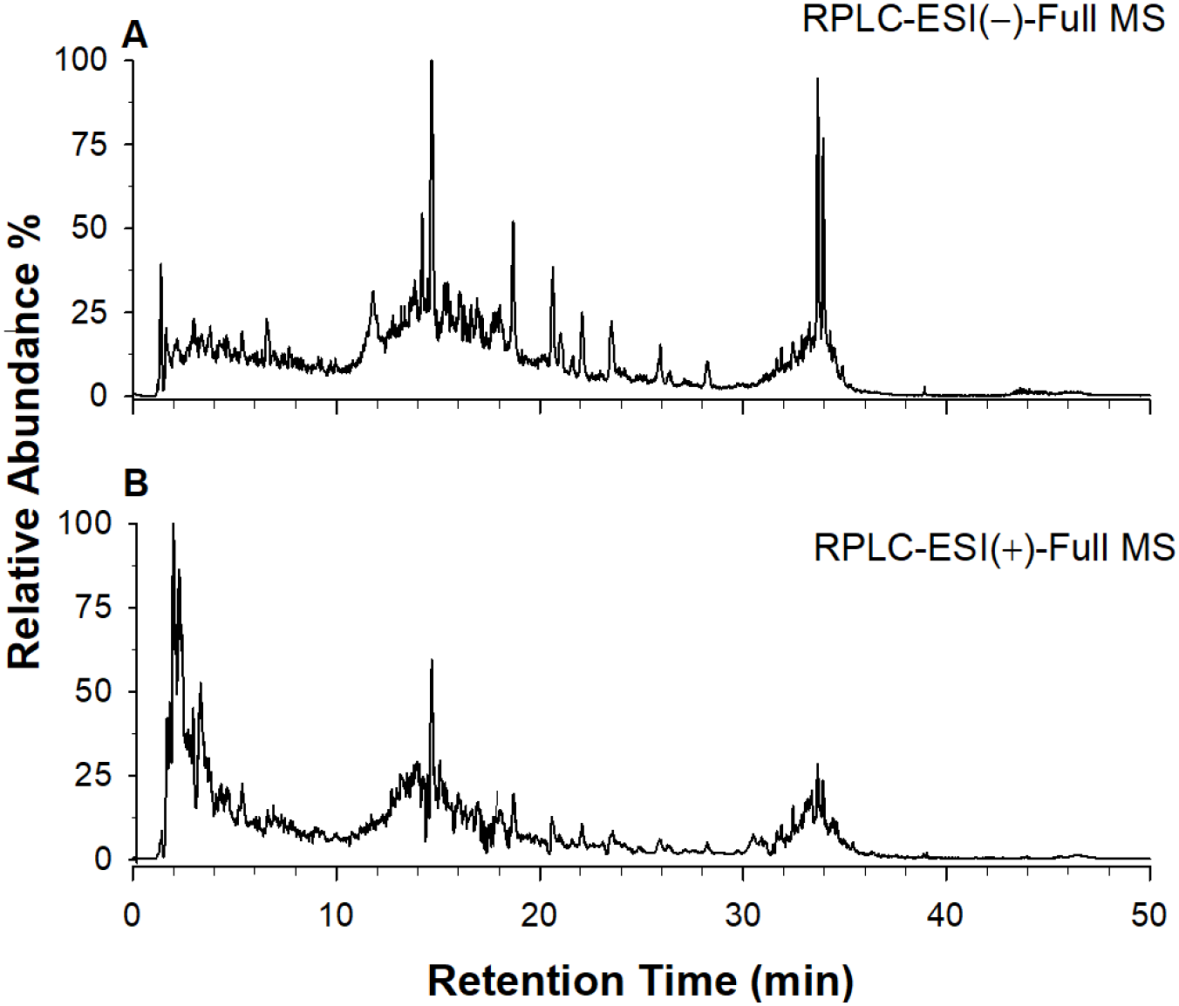
Representative total ion current (TIC) chromatograms in negative **(A)** and positive **(B)** ion mode for biostimulant extracts.

**Figure 2:**
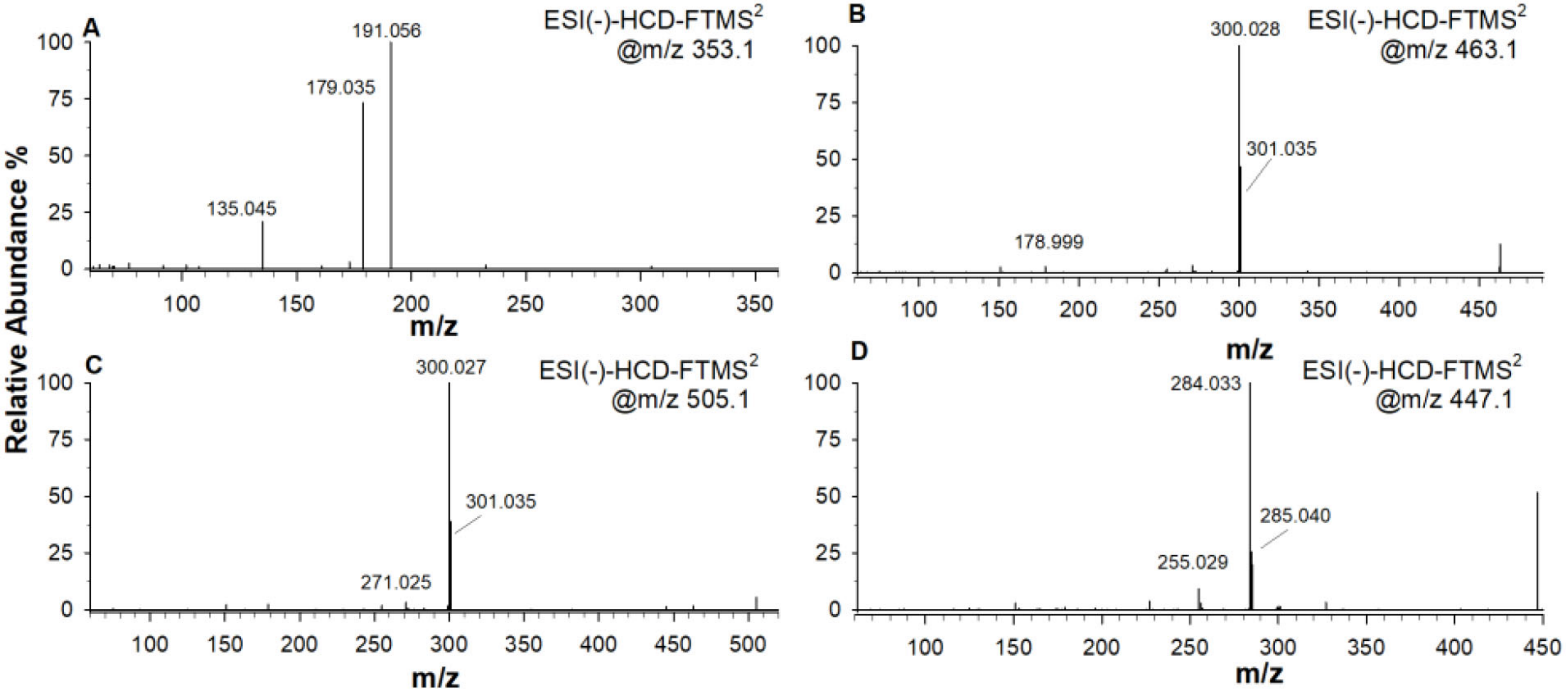
Panel **(A)** shows a tandem mass spectrum of precursor ion isolated at m/z 353.1 with major peak fragments at m/z 135.045, 179.035, and 191.056; panel **(B)** displays MS/MS spectrum of ion at m/z 463.1 with fragment peaks at m/z 178.999, 300.028, and 301.035; panel **(C)** includes the peaks at m/z 271.025, 300.027, and 301.035 arising from ion at m/z 505.1; panel **(D)** features peaks at m/z 255.029, 284.033, and 285.040 from precursor at m/z 447.1. All spectra are labeled ESI(-)-HCD-FTMS² at specific m/z values.

The qualitative analysis of the phenolic composition from biostimulant formulation has been carried out using RPLC-DAD-ESI-MS/MS in negative and positive ionization mode. Representative total ion chromatograms (TIC) relevant to biostimulant extract, in both negative and positive ion mode, are shown in **Figures 1A, B**, respectively. The phenolic compounds were cautiously identified by MS data together with the interpretation of MS/MS and UV spectra in comparison with those found in literature. The certain identities could be obtained after screening of authentic standards. Here, 72 phenolic compounds were tentatively identified in the biostimulant formulation; the identities, retention times, and observed molecular and fragment ions in negative and positive ion polarities of individual compounds are presented in **Table 1**. As an example, **Figure 2** shows the MS/MS spectra in negative mode of compounds 17, 45, 51, 56 reported in **Table 1**.

**Table 1:** Number of compounds, identity, retention time, and observed molecular and fragment ions in negative and positive ion polarities respectively.

Compound I.D.	Name	RT (min)	[M-H]^-^ m/z	MS^2^ neg	[M+H]^+ ^ m/z	MS^2^ pos
1	Turanose	1.26			342.1162	325.113, 145.049, 127.093, 97.029, 85.029, 69.035
2	Fructosyl-lysine	1.42			309.1656	147.114, 128.071, 84.081
3	Mannitol	1.56	181.0718	181.051, 163.040, 135.043, 119.050		
4	Fructose 6- phosphate	1.6	259.0226	187.109, 96.965, 78.959		
5	Quinic acid	1.7	191.0561	173.045, 127.040, 111.087, 85.0298		
6	Ferulic acid derivative	2.0	290.088	254.104, 230.104, 188.093, 146.083		
7	Citric acid	2.06	191.0198	111.009, 87.009		
8	Guanosine-5'- phosphate	2.2	362.0511	211.002, 150.042, 78.959		
9	Gallic acid monohydrate	2.3	187.0243	169.051, 125.025		
10	Gallic acid	2.5	169.0143	125.025, 97.029		
11	O-Methylguanosine	2.8	296.1000	206.068, 150.042, 133.015		
12	Pantothenic acid	4.1	218.1034	146.082, 88.040, 71.014		
13	Protocatechuic acid	4.4	153.0194	109.029, 78.959		
14	Tryptophan	5.45	203.029	186.056, 116.051, 72.009	205.0977	188.070, 159.092, 146.060, 118.065
15	Glucopyranosiduronic acid	5.65	342.1194	208.109, 132.030, 115.004		
16	Dimethyladenosine	6.7			296.1366	296.134, 164.093, 86.097
17	Neochlorogenic acid	7.21	353.0880	191.056, 179.035, 135.045		
18	ϒ-Glutamylleucine	7.3	259.1303	241.119, 130.087, 128.035	[M+NH_4_]^+^ 261.1456	244.117, 198.112, 132.012, 86.097
19	Salicilic acid	7.5	137.0239	93.035		
20	Hydroxybenzoic acid *O-*deoxyhexoside	9.5	283.0852	239.092, 137.025, 93.035		
21	Glutamylphenylalanine	9.7	293.1149	164.078, 128.035	295.1303	278.104, 166.086, 120.081, 84.045
22	p-Coumaroyl quinic acid	10.18	337.0929	191.057, 173.047, 163.040, 119.050		
23	Oglufanide	10.2	332.1261	314.117, 288.135, 270.126, 203.083, 185.057		
24	Chlorogenic acid	10.8	353.088	191.056, 179.035, 135.045		
25	(+)-Catechin	12.53	289.072	289.072, 245.082, 205.051, 203.071, 109.029, 125.024, 179.035, 151.040, 137.024, 123.045, 161.061, 165.019, 221.082, 187.040, 149.024		
26	Cryptochlorogenic acid	12.91	353.088	191.056, 179.035, 173.045, 135.045		
27	Caffeoyl-O-methylquinic acid	12.94 15.57	367.1029	93.035, 173.045, 191.056		
28	Vanilglycolic acid Methoxyisophthalic acid	13.34 16.49	195.0299	165.020, 151.040, 123.045		
29	Golotimod	13.5	332.1270	203.083, 128.035	334.1416	306.061, 278.066, 188.071, 146.060
30	Caffeoyl-deoxyquinic acid	13.8	337.0929	191.056, 173.046,163.041, 119.0503		
31	4-p-Coumaroylquinic acid	14.6	337.0929	173.046, 163.041, 155.036, 137.025		
32	apigenin 6,8-digalactoside	14.7			595.1671	409.091, 379.081, 355.080, 325.070, 295.060
33	Isovitexin -*O*-hexoside	14.8	593.1518	503.121, 473.110, 383.078, 353.067		
34	3-O-Feruloylquinic acid	15.4	367.1044	193.050, 191.056, 173.046, 93.035		
35	Saponarin	15.5	593.1535	503.120, 473.109, 383.078, 353.067, 311.056		
36	Hydrojuglone glucoside	16.1	337.0929	119.056, 163.041		
37	Quercetin 3-gentiobioside	16.1	625.1440	343.026, 301.035, 300.028, 178.999		
38	Orientin	16.4	447.0955	357.062, 327.051, 297.040		
39	Ferulic acid	16.7	193.0507	178.028,149.061 134.038		
40	Schaftoside	16.8	563.1431	545.132, 473.109, 443.099, 383.077, 353.067		
41	Leu-Val-leu	17.12	342.2398	229.156, 130.088		
42	Isoviolanthin	17.7	577.1590	503.120, 457.115, 383.078, 353.067, 300.029		
43	Rutin	18.29	609.146	300.027, 301.035		
44	Vitexin/Isovitexin	18.4	431.2109	353.067, 341.067, 311.057, 283.062, 269.046	433.1155	397.091, 379.081, 337.070, 313.070, 283.059
45	Isoquercetrin/ Hyperoside	18.5	463.0896	300.028, 301.035, 178.999	465.1044	303.049, 127.040, 86.097, 85.029
46	N-lactoyl-phenylalanine	18.6	236.0939	164.073, 147.045, 88.040		
47	Kaempferol 3-O-gentiobioside	19.0	609.1499	327.054, 285.041, 284.033		
48	Quercetin 3-β-D-Glucoside	19.1	463.088	300.027, 301.035		
49	Cyanidin 3-O-Rutinoside	20.5	593.151	284.033, 285.040		
50	Quercetin 3-O-malonylglucoside	20.6			551.1029	303.049, 127.039, 85.029
51	Quercetin *O*-acetyl hexoside	21	505.0987	300.027, 301.035, 271.025		
52	Veronicastroside	21.1	593.1533	327.050, 285.041, 284.033		
53	Allocryptopine	21.4			370.1673	352.154, 290.094, 206.081, 188.070
54	Cynaroside	22	447.0938	327.051, 285.041, 284.033, 255.030, 151.004		
55	Isorhamnetin-3-O-rutinoside	22.3	623.1636	315.051, 314.044, 300.027, 299.019		
56	Kaempferol 6- *C*- hexoside	22.5	447.0930	284.033, 285.040, 255.029		
57	Tamarixetin	23.5	315.0511	300.028, 151.004	317.066	302.042, 285.039, 153.018
58	Isorhamnetin 3-hexoside	23.5	477.1042	357.062, 314.044, 285.041, 271.025, 243.030		
59	Moringyne	26.0	311.113	293.213, 275.202, 223.171, 201.114		
60	Kaempferol *O*-acetyl hexoside	26.4	489.228	327.052, 285.041, 284.033		
61	Ferulic acid derivative	30.3	285.1246	193.038, 175.028, 161.048		
62	Ferulic acid derivative	32.5	301.072	273.041, 193.014, 178.999, 151.004		
63	Quercetin	32.6	301.035	301.035, 273.041, 151.004, 178.999, 121.030	303.050	303.050, 285.039, 257.044, 153.017
64	Kaempferol	22	285.0405	285.040, 267.160, 151.004	287.0554	287.055, 269.045, 153.018
65	Oxo-dihydroxyoctadecenoic	33.6	327.2171	309.207, 291.197, 229.145 211.134, 171.103		
66	Catechin hydrate	34.0	307.1930	289.181, 235.134, 185.118		
67	Trihydroxyoctadecenoic acid	34.3	329.2333	311.223, 229.145, 211.134, 171.103		
68	Phytosphingosine	34.3			318.3004	300.288, 282.278, 270.280, 60.045
69	Sphingosine	34.4			300.2897	282.278, 270.278, 264.268, 254.284, 60.045
70	9-hydroxy-10,12-octadecadienoic acid	35.6	295.2278	277.217, 195.134, 183.139, 171.103		
71	Rugosaflavonoid A	36.6	325.2024	183.012		
72	Kaempferol O-hydroxymethylglutaryl hexoside	38	593.2756	565.533		

The detected metabolites showed that formulation possessed an exceptionally rich and diverse metabolome, dominated by polyphenols, flavonoids, organic acids, amino-acid derivatives, lipids, and bioactive peptides that might function as osmoprotectant. Together, these compounds reflected strong antioxidant capacity, nutritional richness, and metabolic complexity with putative immune-modulating, antimicrobial, and defensive properties (Nephali et al., 2020).

Among phenolic acids and their derivatives, the major recognized compounds were ferulic acid derivatives, p-Coumaroyl quinic acids, neochlorogenic acid, cryptochlorogenic acid, quinic acid, O-caffeoyl-O-methylquinic acid, hydroxybenzoic acid O-deoxyhexoside, salicylic acid, and gallic acid monohydrate, which confirmed high antioxidant potential and a metabolite profile typical of stress-resistant plants (Verma et al., 2009). Neochlorogenic acid is a reported component of *M. oleifera* tissues (Admane et al., 2023), whereas salicylic acid and gallic acid have a well-known relation with the pathways leading to a better resistance to stresses. Particularly salicylic acid plays an important role in the regulation of plant growth, development, ripening, flowering, and responses to abiotic stresses (Miura and Tada, 2014). Gallic acid proved to protect the plant against abiotic stresses such as salt stress (Ozfidan-Konakci et al., 2015) or excessive boron-induced stress, together with benzoic acid and salicylic acid (Farghaly et al., 2021). Chlorogenic acid is associated with beneficial health properties as anti-inflammatory and antimicrobial effects, but it plays also vital roles in resistance mechanisms to abiotic stresses including heavy metal, cold, heat, ultraviolet (UV) light, drought, and salinity, which affect the plant physiological processes, resulting in massive losses of agriculture production (Soviguidi et al., 2022).

Among flavonoids, the detected compounds as C- and O-glycosylated flavones and flavonols, vitexin or isovitexin, orientin, isovitexin-O-hexoside, schaftoside, isoquercitrin or hyperoside, rutin, quercetin and glyco-derivatives, kaempferol derivatives and so, represented the identity markers of bio-extract with very high antioxidant and anti-inflammatory capacity, strong UV-protection and stress-resilience profile (Mukunzi et al., 2011; Goyal et al., 2007). Their formation normally depends on light, so they are mainly concentrated in the outer tissues of the free-standing leaves. The ability of quercetin to induce resistance phenomena in plant tissues has been reported (Sanzani et al., 2010). Kaempferol, in combination with caffeic acid and plant growth-promoting rhizobacteria, was reported to enhance stem length, shoot root, and leaf dry weight, as well as chlorophyll and nutrient uptake thus alleviating salinity stress in potatoes (Ramzan et al., 2024).

Other retrieved compounds include amino acids, dipeptides, tripeptides indicating high nutritional value, protein turnover activity, and the presence of bioactive peptides associated with anti-hypertensive effects, anti-inflammatory activities (Sánchez-MaChado et al., 2010). The role of dipeptides in plant health-promoting activities, including boosting growth and improving stress resilience against phytopathogens, salinity, chilling, and heat, has been reported (Agarwal et al., 2025). A close connection in the enhancement of proline and water deficient tolerance in barley was described, with a positive effect on enzymes and membrane integrity mediating osmotic adjustments under stress (Khan et al., 2020). Organic acids support antioxidant and preservative properties. Small organic acids increased the drought tolerance in different plant species such as spear grass, cotton and tropical grasses (Umezawa et al., 2006) or demonstrate a protective role in oxidative damage under drought stress (Du et al., 2012). While lipid profile provided for known anti-inflammatory lipidome, including bioactive skin-protective sphingoid bases, long-chain amides linked to antimicrobial activity (Fischer, 2020).

### 3.2 Tomato seeds priming assay

**Figure 3:**
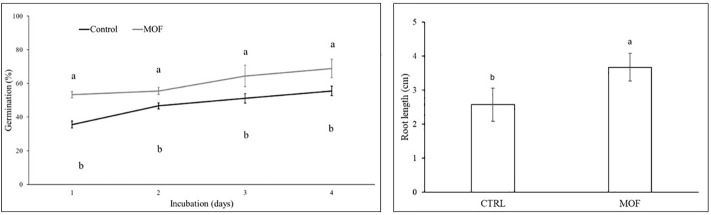
(Left) Germination rate (germinated seeds, %) of tomato seeds primed with the *Moringa oleifera* formulate (MOF). (Right) Root length of germinated seeds expressed in cm. The control consisted of seeds primed with water. For each treatment, data are the average of 3 replicates ± standard error of the mean (SEM). Different letters indicate statistical significance (*p* < 0.05).

For most vegetable crops the cultivation cycle begins with direct seed sowing. As such, in the present investigation we tested the effect of MOF on seed germination (**Figure 3A**). The cultivar used for germination assays was selected for its uniform and rapid germination, enabling reliable assessment of germination parameters. Seeds treated with MOF since the first assessment (24 h) showed a significant faster germination rate than the control (+50%), which remained in the range +19-26% for the entire assessment period (4 days). Homogeneous germination and high seedling vigour could positively influence a productive crop establishment and performance, moreover a fast and vigorous seedlings emerge might increase the chance to capture nutrients, tolerate stresses, compete with weeds, and, overall, deal better with adverse environmental conditions (Cardarelli et al., 2022). The root length was also measured. The germinated seeds showed roots 43% longer than the control seeds (**Figure 3B**), this could allow a better nutrient absorption.

### 3.3 *In planta* assay

#### 3.3.1 Soil analysis

Soil texture plays a key role in regulating water retention, drainage, and root–soil interactions, all of which influence plant responses to drought stress. The chemical and texture analysis conducted to identify hydrological constants and set up the irrigation plan for stressed and non-stressed plants are reported in **Table 2**. The soil resulted composed mainly of clay, followed by silt, and lastly by sand.

**Table 2:** Results of soil analysis.

Soil parameter	Result
Sand	25.7%
Silt	33.9%
Clay	40.4%
Texture	Clay
pH in water	8.07
pH in KCl	7.43
Electrical conductivity	151.4 μS cm^-1^
Water content between field capacity and wilting point	10.23% by volume of soil

**Figure 4:**
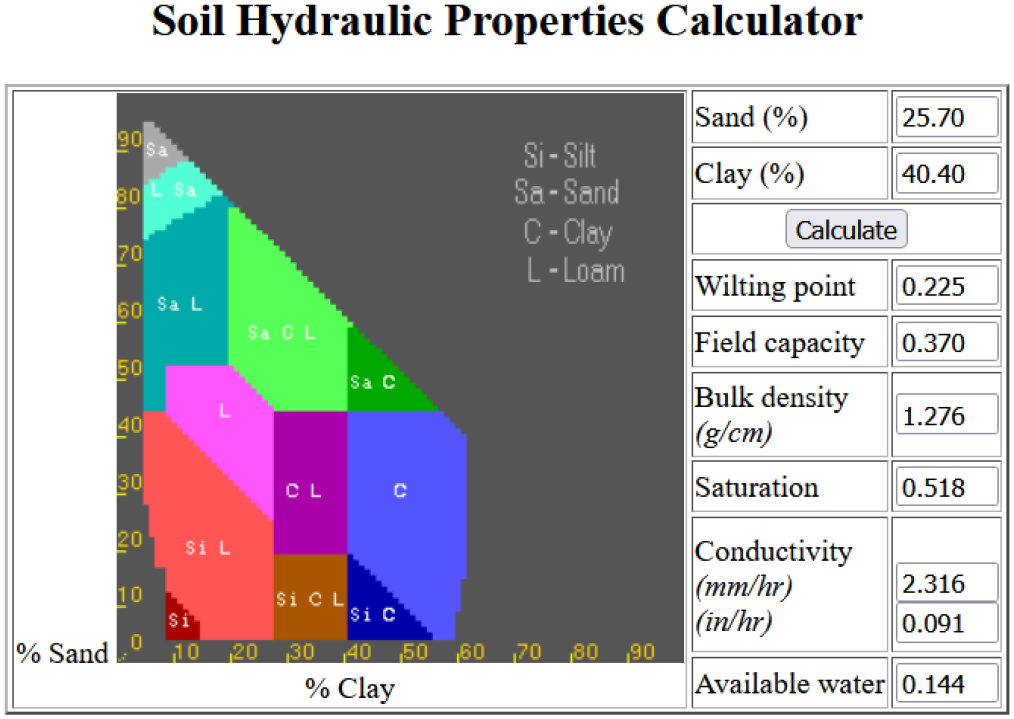
Calculation sheet for hydrological constants. http://www.dynsystem.com/netstorm/soilwater.html.

The hydrological constants were detected by incorporating the particle size distribution data into a calculation model as reported in **Figure 4**.

#### 3.3.2 Vegetative parameters

**Figure 5:**
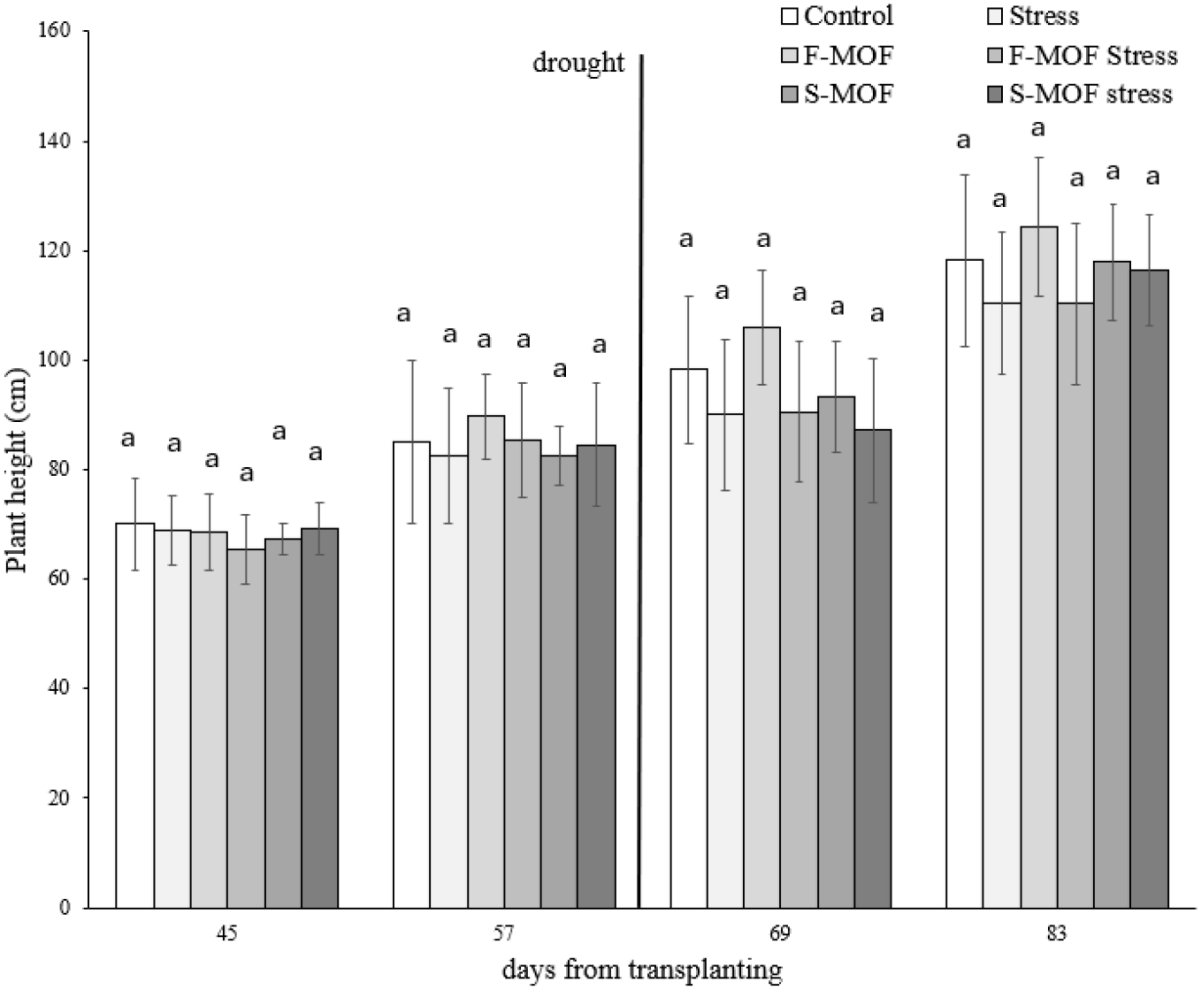
Effect of MOF applied as spray on leaves (F-MOF) or as drenching to soil (S-MOF) on the height (cm) of tomato plants in absence and presence of water stress (F-MOF stress/S-MOF stress). Stressed and non-stressed untreated plants were used as controls. Data are the average of 15 replicates ± standard error of the mean (SEM). Different letters indicate statistical significance (p < 0.05).

The tomato cultivar used for growth assays was known to be well characterized for vegetative growth under controlled conditions. To assess the efficacy of the formulation on the plant growth before and after the stress, the height of the plants was measured at 45, 57, 69, and 83 days from the transplant (**Figure 5**). In absence of the stress (up to 57^th^ day), the plants treated by MOF foliar application (F-MOF) showed an increasing trend in plant height, although the differences were not statistically significant. In plants subjected to water stress (from the 69^th^ day), MOF applied by drenching (S-MOF) induced an increasing trend in plant height, when compared to the stressed control, although also in this case the result was not supported by statistical evidence. This trend in plant height is consistent with observed changes in other growth-related parameters, supporting a coordinated growth response.

**Figure 6:**
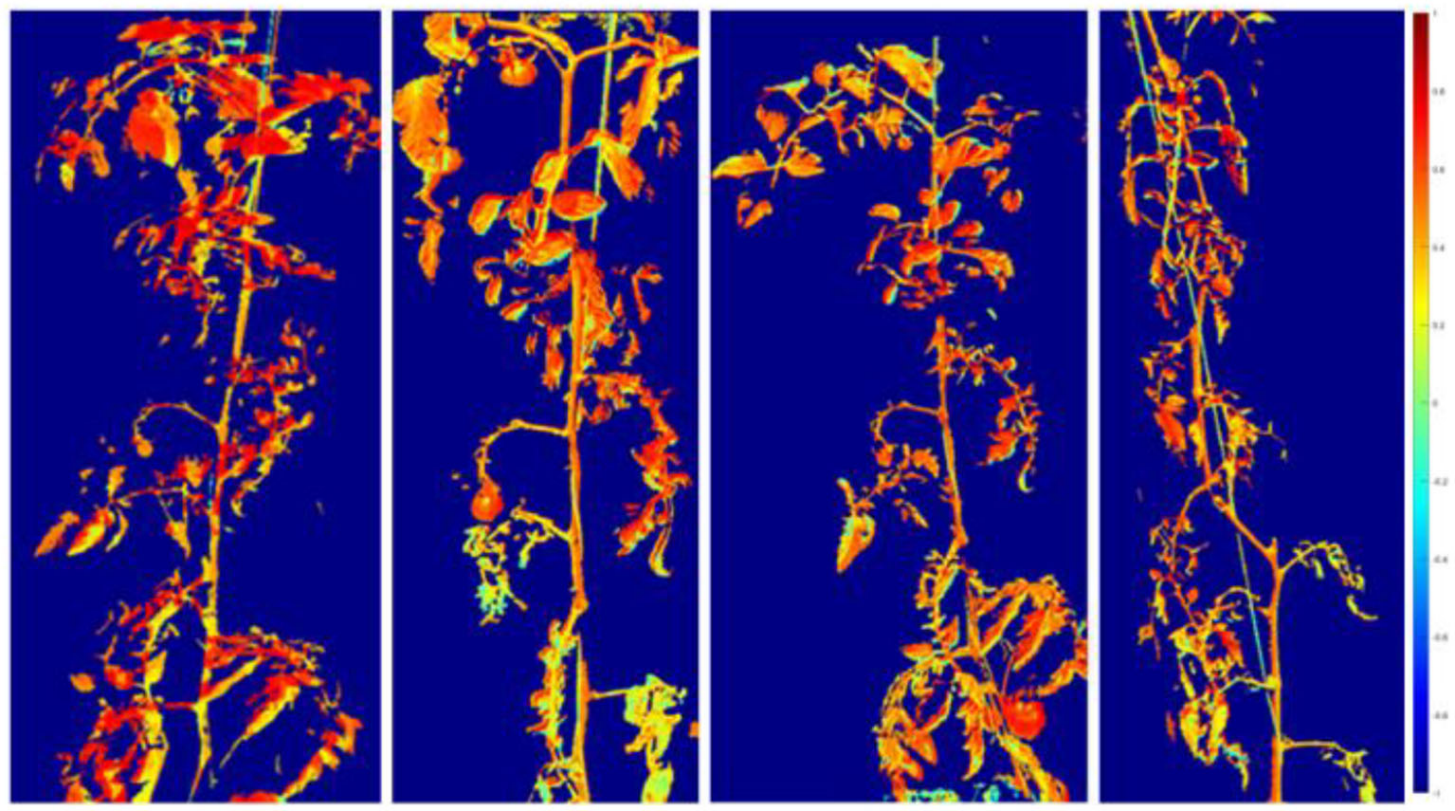
Normalized Difference Vegetation Index (NDVI) images of a stressed tomato plant on days 0, 3, 7, and 11 of drought application.

**Figure 7:**
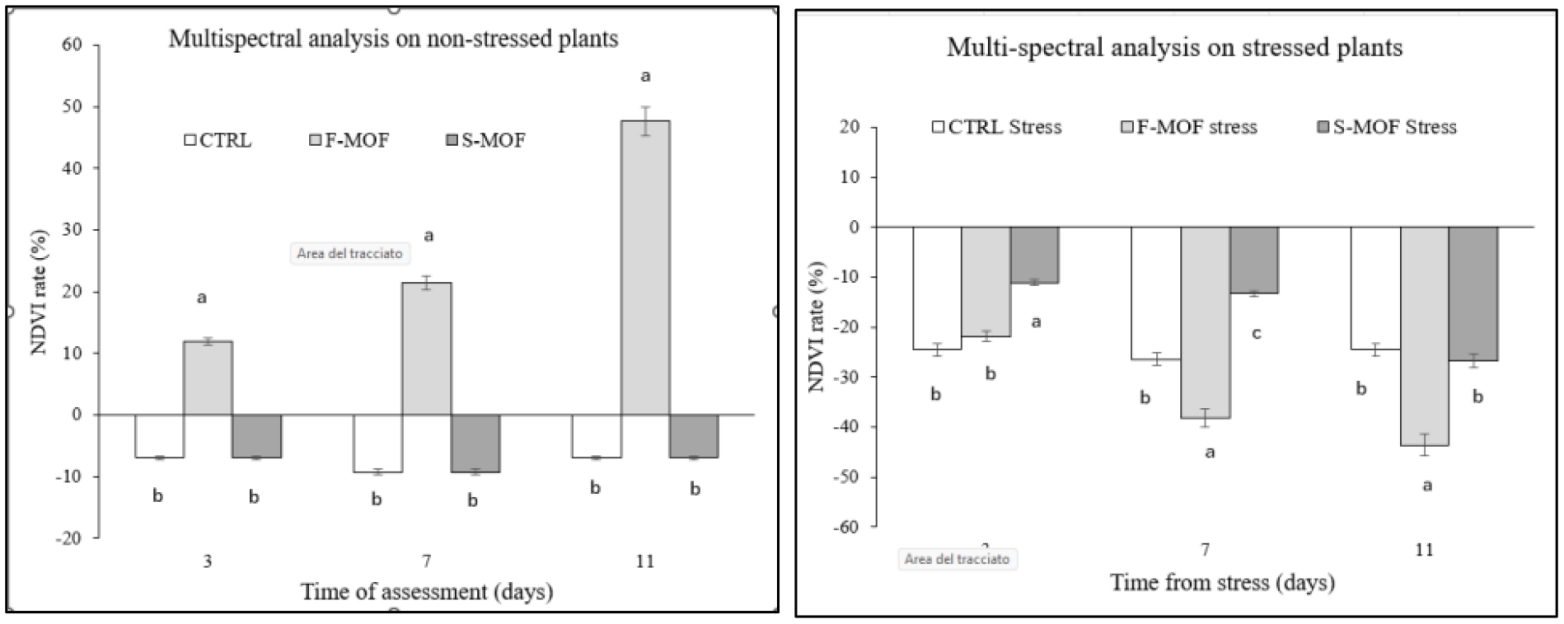
Normalized difference vegetation index (NDVI) rate (%) following MOF foliar (F-) and soil (S-) application on non-stressed (left) and stressed (right) tomato plants as compared to the untreated control. Data are the average of 3 replicates ± standard error of the mean (SEM). For each time point, different letters indicate statistical significance (*p* < 0.05).

The vegetative vigour of the plants was also assessed by multispectral measurements performed on days 0, 3, 7, and 11 of the water stress on both stressed and non-stressed plants. The NDVI plant mask of three plants for each treatment was obtained after aligning the different multispectral bands of the acquired images, which allowed the monitoring of the progress of water stress through the decrease in the brightness of green and leaf surface area (**Figure 6**). In absence of stress (**Figure 7A**), it was possible to observe that the NDVI rate of plants treated with MOF distributed by foliar spray (F-MOF) increased as compared to the soil treatment (S-MOF) and to the untreated control, which showed a decreasing trend. As such, the foliar application seemed to have a stimulating effect on the vegetative parameters, as observed also in plant hight as a trend. This could be ascribed to a faster and more efficient absorption of bioactive molecules through the aerial apparatus as also observed by Suchithra et al. (2022). The positive outcome of nutrients’ application by foliar spray is the result of the selection of a proper dose as a compromise between efficacy and lack of phytotoxicity. However, a plethora of factors such as temperature, light intensity, humidity, might play a key role in the efficiency of foliar applications (Fernández and Eichert, 2009).

When stressed plants were considered (**Figure 7B**), the tolerance to drought stress was significantly higher in plants treated with MOF distributed to the soil by drenching (S-MOF-stress) as compared to the control up to 7^th^ day of stress application; however, when the stress continued, this initial effect was lost (11^th^ day). These results confirmed those obtained by other parameters as the trend in plant height (**Figure 5**) and support the effectiveness of MOF as a resistance inducer to drought. Indeed, the drenching could ameliorate the nutrient absorption through the root system, thus counteracting the shortage in the water/nutrient availability. This hypothesis is supported by the increase in root length following MOF application observed in germination assay. For example, hydrolysates could form complexes and chelates between soil micronutrients such as Zn, Fe, Cu and Mn, thereby aiding in nutrient availability and uptake in the roots (Colla and Rouphael, 2015).

#### 3.3.3 Determination of pigment content

**Figure 8:**
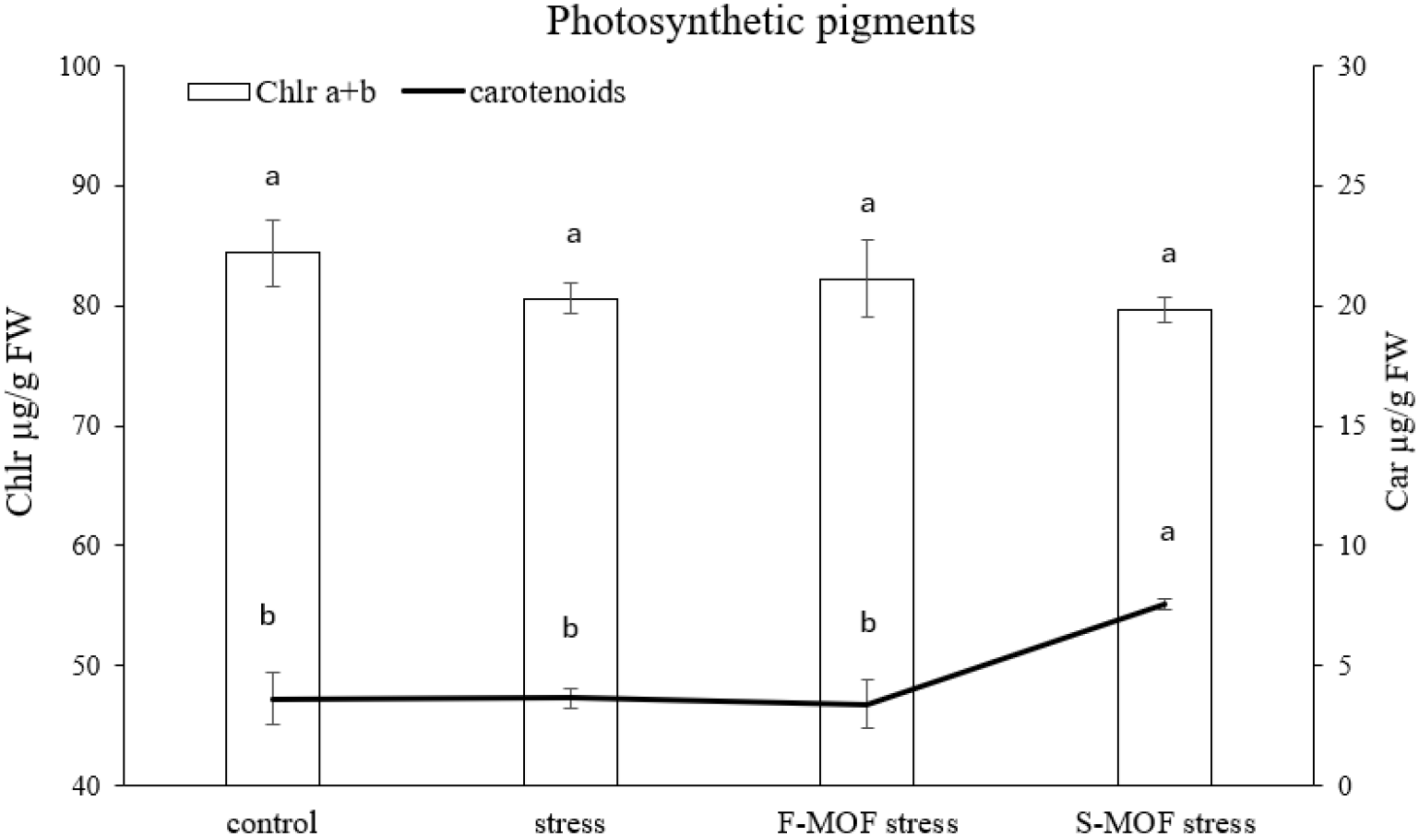
Total chlorophyll and carotenoids content following MOF foliar (F-) and soil (S-) application on stressed tomato plants. Data are the average of 3 replicates ± standard error of the mean (SEM). Different letters indicate statistical significance (*p* < 0.05).

The results of assay on total chlorophyll and carotenoids are reported in **Figure 8**. The assessment was conducted on leaves harvested on the 7^th^ day of drought stress as the time point at which the highest differences among treatments were recorded (**Figure 7B**). MOF treatments did not increase chlorophyll content as compared to untreated stressed plants. However, a significant increase in the carotenoid content was observed in plants treated by MOF as soil treatment (S-MOF, +75%). The reduction of chlorophyll content is a physiological effect, typical of plants under stress conditions, like infection by pathogens and abiotic stresses, such as drought, high salinity, temperature variations, lack of minerals and nutrients (Othman, 2009). In the present investigation no significant reduction in chlorophyll content was observed in drought-stressed plants. This suggests that the applied water deficit did not cause structural damage to the photosynthetic apparatus. Indeed, drought effects at the fruit set stage may primarily involve stomatal limitation and physiological acclimation, allowing plants to maintain chlorophyll levels to support ongoing carbon assimilation (Sun et al., 2023; Zhuang et al., 2020; Ben Sedrine et al., 2024). While chlorophyll content reflects the structural stability of photosynthetic machinery, carotenoids act as flexible photoprotective pigments that are readily upregulated in response to stress and external stimuli. Carotenoids, being accessory pigments, have an important role of protection of photosynthetic apparatus against ROS generation, especially from singlet oxygen (^1^O_2_) produced by triplet excited chlorophylls (^3^Chl*) (Ramel et al., 2013; Swapnil et al., 2021). One of the main carotenoids with a detoxifying role is β-carotene, which acts as a precursor of strigolactones (SL), an important class of phytohormones involved in plant growth and development (Al-Babili & Bouwmeester, 2015). This might explains why stressed plants with a higher carotenoid content (S-MOF) had the best growth trend and NDVI results. β-carotene is also a precursor of zeaxanthin and violaxanthin, precursors to abscisic acid, a key hormone in stress response and stomatal closure (Moreno et al., 2021). Drought stress is known to enhance ROS production due to imbalances between light absorption and carbon assimilation (Hasanuzzaman et al., 2021). In this study, biostimulant application by drenching significantly increased carotenoid content in stressed plants, which might be associated with altered ROS accumulation. Carotenoid accumulation may therefore contribute in preventing oxidative damage to the photosynthetic apparatus, consistent with the observed maintenance of chlorophyll content under drought stress.

#### 3.3.4 Leaf relative water content

**Figure 9:**
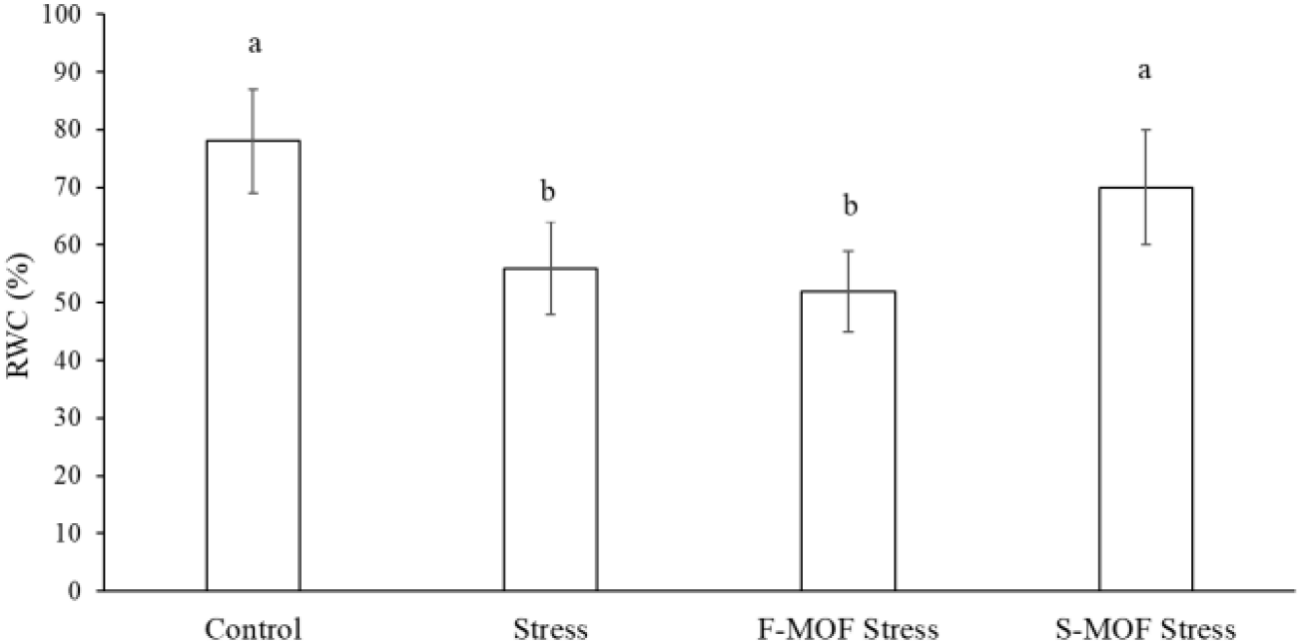
Leaf relative water content (RWC, %) following MOF foliar (F-) and soil (S-) application on stressed tomato plants. Data are the average of 3 replicates ± standard error of the mean (SEM). Different letters indicate statistical significance (*p* < 0.05).

The relative water content (RWC, %) was determined on leaves harvested during the last day of drought stress (day 11). In plant treated by S-MOF, a RWC value higher than that of the stressed control (+25%) and similar to the one of the non-stressed control was recorded; whereas the RWC value of F-MOF treated plants was similar to that of the stressed plant and lower than that of the non-stressed control (**Figure 9**). Abd El-Mageed et al. (2016) proved that there is a strong relationship between the relative water content and plant biomass under the combined effect.

#### 3.3.5 Production parameters

**Figure 10:**
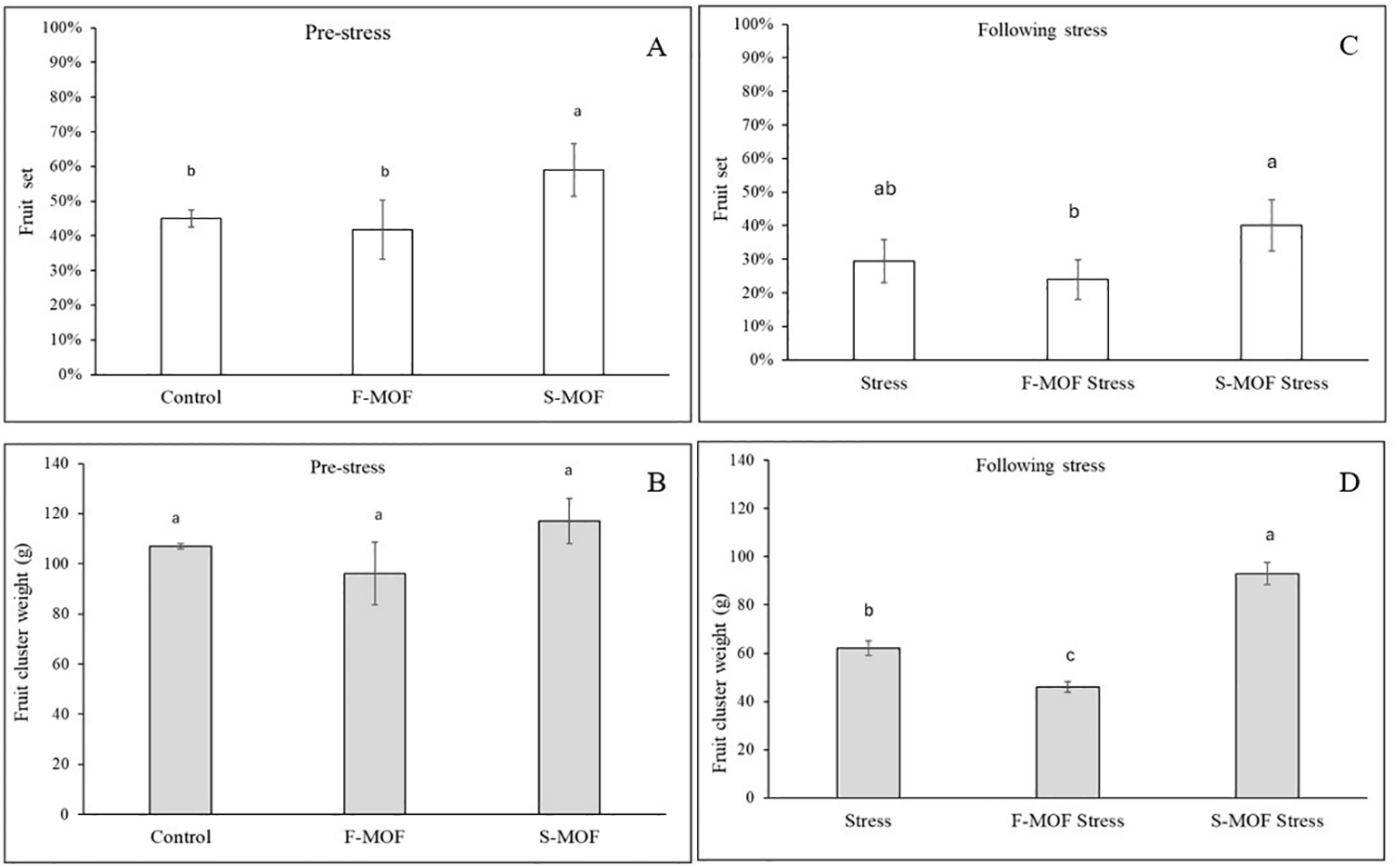
Fruit set (%) and fruit cluster weight (g) following MOF foliar (F-) and soil (S-) application on non-stressed **(A, B)** and stressed **(C, D)** tomato plants. Data are the average of 15 replicates ± standard error of the mean (SEM). Different letters indicate statistical significance (*p* < 0.05).

To evaluate the effectiveness of the formulation on plant production, the fruit set and the weight of the fruit clusters were considered. The fruits received the drought stress during fruit set phase of the second flowering. In absence of water stress (first flowering), the percentage of fruit set was higher (+9%) in the plants treated with the formulate applied to the soil (S-MOF, **Figure 10A**), whereas concerning fruit cluster weight, S-MOF performed similarly to the control (**Figure 10B**). This finding was confirmed and even emphasised in stressed plants (**Figures 10C, D**). These results could be ascribed to the induction of different morphological, physiological, biochemical, and molecular mechanisms. Similarly, it has been reported that the application of a *Moringa* leaf extract to mandarin plants increased fruit set, quality, and quantity and reduced fruit drop (Nasir et al., 2016). A *Moringa* leaf extract applied to plums increased the quality and quantity as well as the antioxidant activity of fruit (Thanaa et al., 2017).

## 4 Conclusions

This study showed that the tested formulation had different effects depending on the application strategy and the plant conditions. *M. oleifera* formulate proved to be an effective biostimulant when applied through foliar sprays in the absence of stress, resulting in an enhanced vegetative status of the plants. These results suggest that this formulation could be successfully applied in leafy vegetables, which are usually grown from autumn to spring, when water stress is generally less frequent. However, *M. oleifera* formulate effect on plants under stress conditions was better expressed when applied as soil drenching. Therefore, this use is recommended in summer crops subjected to adverse climatic conditions (*e.g.* drought). Furthermore, the formulation applied to the soil proved to successfully increase fruit set without compromising fruit cluster weight in tomato plants subjected to drought stress, making it particularly suitable for early summer crops such as greenhouse-grown zucchini and tomato. Although further studies on a larger scale are needed, the results obtained in this work support the tested *M. oleifera* formulate as effective biostimulant, capable to “stimulate” plant primary and secondary metabolism, including the response to stress.

## Data Availability

The original contributions presented in the study are included in the article/supplementary material. Further inquiries can be directed to the corresponding author.

## References

[B1] Abd El-Mageed T. A. Semida W. M. Mohamed G. F. Rady M. M. (2016). Combined effect of foliar-applied salicylic acid and deficit irrigation on physiological–anatomical responses, and yield of squash plants under saline soil. South Afr. J. Bot. 106, 8–16. doi: 10.1016/j.sajb.2016.05.005, PMID: 41788901

[B2] Admane N. Cavallo G. Hadjila C. Cavalluzzi M. M. Rotondo N. P. Salerno A. . (2023). Biostimulant formulations and moringa oleifera extracts to improve yield, quality, and storability of hydroponic lettuce. Molecules 28, 373. doi: 10.3390/molecules28010373, PMID: 36615566 PMC9822398

[B3] Agarwal P. Fischer H. D. Camalle M. D. Skirycz A. (2025). Not to be overlooked: dipeptides and their role in plant stress resilience. J. Exp. Bot. 76, 5738–5747. doi: 10.1093/jxb/eraf311, PMID: 40628532 PMC12516527

[B4] Al-Babili S. Bouwmeester H. J. (2015). Strigolactones, a novel carotenoid-derived plant hormone. Annu. Rev. Plant Biol. 66, 161–186. doi: 10.1146/annurev-arplant-043014-114759, PMID: 25621512

[B5] Ben Sedrine I. Werghi S. Hachef A. Maalaoui A. Zarkouna R. Akriche S. . (2024). Alleviation of drought stress in tomato by foliar application of seafood waste extract. Sci. Rep. 14, 30572. doi: 10.1038/s41598-024-80798-0, PMID: 39706919 PMC11662016

[B6] Bergougnoux V. (2014). The history of tomato: From domestication to biopharming. Biotechnol. Adv. 32, 170–189. doi: 10.1016/j.biotechadv.2013.11.003, PMID: 24211472

[B7] Bulgari R. Franzoni G. Ferrante A. (2019). Biostimulants application in horticultural crops under abiotic stress conditions. Agronomy 9, 306. doi: 10.3390/agronomy9060306, PMID: 41725453

[B8] Calvo P. Nelson L. Kloepper J. W. (2014). Agricultural uses of plant biostimulants. Plant Soil 383, 3–41. doi: 10.1007/s11104-014-2131-8, PMID: 41788969

[B9] Cardarelli M. Woo S. L. Rouphael Y. Colla G. (2022). Seed treatments with microorganisms can have a biostimulant effect by influencing germination and seedling growth of crops. Plants 11, 259. doi: 10.3390/plants11030259, PMID: 35161239 PMC8838022

[B10] Ceccarelli A. V. Miras-Moreno B. Buffagni V. Senizza B. Pii Y. Cardarelli M. . (2021). Foliar application of different vegetal-derived protein hydrolysates distinctively modulates tomato root development and metabolism. Plants 10, 326. doi: 10.3390/plants10020326, PMID: 33567668 PMC7914860

[B11] Colla G. Rouphael Y. (2015). Biostimulants in horticulture. Sci. Hortic. 196, 1–2. doi: 10.1016/j.scienta.2015.10.044, PMID: 41788901

[B12] da Silva Monteiro L. I. Carvalho A. J. D. B. A. Magnani M. do Santos Lima M. (2025). Salting-out liquid-liquid extraction (SALLE) of phenolic compounds of fruits and ultra-fast RP-HPLC/DAD analysis: Validation of the method and characterization of fruits from the Brazilian Caatinga biome. J. Food Composition Anal. 48(1), 108214. doi: 10.1016/j.jfca.2025.108214, PMID: 41788901

[B13] Du J. Qi J. Wang D. Tang Z. (2012). Facile synthesis of Au@TiO 2 core–shell hollow spheres for dye-sensitized solar cells with remarkably improved efficiency. Energy Environ. Sci. 5, 6914–6918. doi: 10.1039/c2ee21264a, PMID: 41788032

[B14] European Biostimulants Industry Council (EBIC) (2025). Regulatory. Available online at: https://biostimulants.eu/regulatory/ (Accessed 1 July 2025).

[B15] European Commission (2019). Regulation (EU) 2019/1009 of the European Parliament and of the Council of 5 June 2019 laying down rules on the making available on the market of EU fertilising products and amending Regulations (EC) No 1069/2009 and (EC) No 1107/2009 and repealing Regulation (EC) No 2003/2003. Available online at: http://data.europa.eu/eli/reg/2019/1009/oj (Accessed September 8, 2025).

[B16] FAOSTAT (2023). Crops and livestock products. Available online at: https://www.fao.org/faostat/en/data (Accessed July 1, 2025).

[B17] Farghaly F. A. Salam H. K. Hamada A. M. Radi A. A. (2021). The role of benzoic acid, gallic acid and salicylic acid in protecting tomato callus cells from excessive boron stress. Scientia Hortic. 278, 109867. doi: 10.1016/j.scienta.2020.109867, PMID: 41788901

[B18] Fernández V. Eichert T. (2009). Uptake of hydrophilic solutes through plant leaves: current state of knowledge and perspectives of foliar fertilization. Crit. Rev. Plant Sci. 28, 36–68. doi: 10.1080/07352680902743069, PMID: 41783271

[B19] Ferreira P. M. P. Farias D. F. Oliveira J. T. D. A. Carvalho A. D. F. U. (2008). Moringa oleifera: bioactive compounds and nutritional potential. Rev. Nutrição 21, 431–437. doi: 10.1590/S1415-52732008000400007, PMID: 41779527

[B20] Fischer C. L. (2020). Antimicrobial activity of host-derived lipids. Antibiotics 9, 75. doi: 10.3390/antibiotics9020075, PMID: 32054068 PMC7168235

[B21] Francesca S. Cirillo V. Raimondi G. Maggio A. Barone A. Rigano M. M. (2021). A novel protein hydrolysate-based biostimulant improves tomato performances under drought stress. Plants 10, 783. doi: 10.3390/plants10040783, PMID: 33923424 PMC8073256

[B22] Goyal B. R. Agrawal B. Goyal R. K. Mehta A. A. (2007). Phyto-pharmacology of *Moringa oleifera* Lam.o An overview. Nat. Prod Radiance 6, 347–353.

[B23] Hasanuzzaman M. Parvin K. Bardhan K. Nahar K. Anee T. I. Masud A. A. C. . (2021). Biostimulants for the regulation of reactive oxygen species metabolism in plants under abiotic stress. Cells 10, 2537. doi: 10.3390/cells10102537, PMID: 34685517 PMC8533957

[B24] Kadoglidou K. I. Anthimidou E. Krommydas K. Papa E. Karapatzak E. Tsivelika N. . (2025). Effect of biostimulants on drought tolerance of greenhouse-grown tomato. Horticulturae 11, 601. doi: 10.3390/horticulturae11060601, PMID: 41725453

[B25] Kauffman G. L. Kneivel D. P. Watschke T. L. (2007). Effects of a biostimulant on the heat tolerance associated with photosynthetic capacity, membrane thermostability, and polyphenol production of perennial ryegrass. Crop Sci. 47, 261–267. doi: 10.2135/cropsci2006.03.0171

[B26] Khan N. Ali S. Zandi P. Mehmood A. Ullah S. Ikram M. . (2020). Role of sugars, amino acids and organic acids in improving plant abiotic stress tolerance. Pak. J. Bot. 52, 355–363. doi: 10.30848/PJB2020-2(24), PMID: 41791163

[B27] Mashamaite C. V. Ngcobo B. L. Manyevere A. Bertling I. Fawole O. A. (2022). Assessing the usefulness of Moringa oleifera leaf extract as a biostimulant to supplement synthetic fertilizers: A Review. Plants 11, 2214. doi: 10.3390/plants11172214, PMID: 36079596 PMC9459878

[B28] Mitchell V. D. Taylor C. M. Bauer S. (2014). Comprehensive analysis of monomeric phenolics in dilute acid plant hydrolysates. Bioenergy Res. 7, 654–669. doi: 10.1007/s12155-013-9392-6, PMID: 41788969

[B29] Miura K. Tada Y. (2014). Regulation of water, salinity, and cold stress responses by salicylic acid. Front. Plant Sci. 5, 4. doi: 10.3389/fpls.2014.00004, PMID: 24478784 PMC3899523

[B30] Moreno J. C. Mi J. Alagoz Y. Al-Babili S. (2021). Plant apocarotenoids: from retrograde signaling to interspecific communication. Plant J. 105, 351–375. doi: 10.1111/tpj.15102, PMID: 33258195 PMC7898548

[B31] Mukunzi D. Nsor-Atindana J. Xiaoming Z. Gahungu A. Karangwa E. Mukamurezi G. . (2011). Comparison of volatile profile of Moringa oleifera leaves from Rwanda and China using HS-SPME. Pakistan J. Nutr. 10, 602–608. doi: 10.3923/pjn.2011.602.608

[B32] Mullan D. Pietragalla J. J. P. A. (2012). Leaf relative water content. Physiol. Breed. II: A Field guide to wheat phenotyping 25, 25–35.

[B33] Nasir M. Khan A. S. Basra S. A. Malik A. U. (2016). Foliar application of moringa leaf extract, potassium and zinc influence yield and fruit quality of ‘Kinnow’mandarin. Scientia Hortic. 210, 227–235. doi: 10.1016/j.scienta.2016.07.032, PMID: 41788901

[B34] Nephali L. Piater L. A. Dubery I. A. Patterson V. Huyser J. Burgess K. . (2020). Biostimulants for plant growth and mitigation of abiotic stresses: A metabolomics perspective. Metabolites 10, 505. doi: 10.3390/metabo10120505, PMID: 33321781 PMC7764227

[B35] Nuruddin M. M. Madramootoo C. A. Dodds G. T. (2003). Effects of water stress at different growth stages on greenhouse tomato yield and quality. HortScience 38, 1389–1393. doi: 10.21273/HORTSCI.38.7.1389

[B36] Oancea F. Velea S. Fãtu V. Mincea C. Ilie L. (2013). Micro-algae based plant biostimulant and its effect on water stressed tomato plants. Rom. J. Plant Prot 6, 104–117.

[B37] Othman R. (2009). *Biochemistry and genetics of carotenoid composition in potato tubers*. (PA, USA: Lincoln University).

[B38] Ozfidan-Konakci C. Yildiztugay E. Kucukoduk M. (2015). Protective roles of exogenously applied gallic acid in Oryza sativa subjected to salt and osmotic stresses: effects on the total antioxidant capacity. Plant Growth Regul. 75, 219–234. doi: 10.1007/s10725-014-9946-4, PMID: 41788969

[B39] Pusta M. G. Macusi E. S. (2024). Moringa (Moringa oleifera Lam.) leaf extract as biostimulant to enhance growth and yield of bitter gourd (Momordica charantia L.). Davao Res. J. 15, 87–97. doi: 10.59120/drj.v15iNo.2.194

[B40] Qin A. Ning D. Liu Z. Sun B. Zhao B. Xiao J. . (2018). Insentek sensor: An alternative to estimate daily crop evapotranspiration for maize plants. Water 11, 25. doi: 10.3390/w11010025, PMID: 41725453

[B41] Ramel F. Mialoundama A. S. Havaux M. (2013). Nonenzymic carotenoid oxidation and photooxidative stress signalling in plants. J. Exp. Bot. 64, 799–805. doi: 10.1093/jxb/ers223, PMID: 22915744

[B42] Ramzan M. Haider S. T. A. Hussain M. B. Ehsan A. Datta R. Alahmadi T. A. . (2024). Potential of kaempferol and caffeic acid to mitigate salinity stress and improving potato growth. Sci. Rep. 14, 21657. doi: 10.1038/s41598-024-72420-0, PMID: 39294197 PMC11410995

[B43] Rouphael Y. Colla G. (2020). Toward a sustainable agriculture through plant biostimulants: from experimental data to practical applications. Agronomy 10, 1461. doi: 10.3390/agronomy10101461, PMID: 41725453

[B44] Sánchez-MaChado D. I. Núñez-Gastélum J. A. Reyes-Moreno C. Ramírez-Wong B. López-Cervantes J. (2010). Nutritional quality of edible parts of Moringa oleifera. Food analytical Methods 3, 175–180. doi: 10.1007/s12161-009-9106-z, PMID: 41788969

[B45] Sanzani S. M. Schena L. De Girolamo A. Ippolito A. González-Candelas L. (2010). Characterization of genes associated with induced resistance against Penicillium expansum in apple fruit treated with quercetin. Postharvest Biol. Technol. 56, 1–11. doi: 10.1016/j.postharvbio.2009.11.010, PMID: 41788901

[B46] Sims D. Gamon J. (2003). Estimation of vegetation water content and photosynthetic tissue area from spectral reflectance: A comparison of indices based on liquid water and chlorophyll absorption features. Remote Sens. Environment. 84, 526–537. doi: 10.1016/S0034-4257(02)00151-7, PMID: 41737640

[B47] Soviguidi D. R. J. Pan R. Liu Y. Rao L. Zhang W. Yang X. (2022). Chlorogenic acid metabolism: The evolution and roles in plant response to abiotic stress. Phyton 91, 239. doi: 0.32604/phyton.2022.018284

[B48] Suchithra M. R. Muniswami D. M. Sri M. S. Usha R. Rasheeq A. A. Preethi B. A. . (2022). Effectiveness of green microalgae as biostimulants and biofertilizer through foliar spray and soil drench method for tomato cultivation. South Afr. J. Bot. 146, 740–750. doi: 10.1016/j.sajb.2021.12.022, PMID: 41788901

[B49] Sun H. Shi Q. Liu N. Y. Zhang S. B. Huang W. (2023). Drought stress delays photosynthetic induction and accelerates photoinhibition under short-term fluctuating light in tomato. Plant Physiol. Biochem. 196, 152–161. doi: 10.1016/j.plaphy.2023.01.044, PMID: 36706694

[B50] Swapnil P. Meena M. Singh S. K. Dhuldhaj U. P. Marwal A. (2021). Vital roles of carotenoids in plants and humans to deteriorate stress with its structure, biosynthesis, metabolic engineering and functional aspects. Curr. Plant Biol. 26, 100203. doi: 10.1016/j.cpb.2021.100203, PMID: 41788901

[B51] Thanaa S. Kassim N. E. AbouRayya M. S. Abdalla A. M. (2017). Influence of foliar application with moringa (Moringa oleifera L.) leaf extract on yield and fruit quality of Hollywood plum cultivar. J. Hortic. 4, 1–7. doi: 10.4172/2376-0354.1000193, PMID: 39887974

[B52] Umezawa T. Fujita M. Fujita Y. Yamaguchi-Shinozaki K. Shinozaki K. (2006). Engineering drought tolerance in plants: discovering and tailoring genes to unlock the future. Curr. Opin. Biotech. 17, 113–122. doi: 10.1016/j.copbio.2006.02.002, PMID: 16495045

[B53] Varun K. Lakshmi B. S . (2025). “ Understanding stress-Related genes and the role of biostimulants in biotic and abiotic stress response to improve plant breeding and cultivation methods,” in Plant Stress Tolerance ( CRC Press), 151–202.

[B54] Verma A. R. Vijayakumar M. Mathela C. S. Rao C. V. (2009). *In vitro* and *in vivo* antioxidant properties of different fractions of Moringa oleifera leaves. Food Chem. Toxicol. 47, 2196–2201. doi: 10.1016/j.fct.2009.06.005, PMID: 19520138

[B55] Zhuang J. Wang Y. Chi Y. Zhou L. Chen J. Zhou W. . (2020). Drought stress strengthens the link between chlorophyll fluorescence parameters and photosynthetic traits. PeerJ 8, e10046. doi: 10.7717/peerj.10046, PMID: 33024649 PMC7520092

